# Book Notices

**Published:** 1885-07

**Authors:** 


### BOOK NOTICES.

Progressive Medicine. A Scientific and Practical Treatise on Diseases of the Digestive Organs, etc. By Ciro de Suzzara-Verdi, M. D. Pp. 349. Philadelphia: F. E. Bœricke. 1885.

It is a very trite saying that discoveries, new inventions, or dogmas are at first met by opposition, incredulity, and perhaps by ridicule and scorn. This may, unfortunately, be true, but it does not follow that every discovery or invention that is laughed at and ridiculed is thereby treated unjustly. Many merit nothing better! These comments are suggested by the following paragraph from the Preface of Dr. Verdi's *Progressive Medicine:* "It is needless to remark that he who dares to differ and predicate new discoveries [!!] or new thoughts must be ready to meet fearlessly the indignation and contempt of those who think that there is nothing beyond what they know and teach daily."

We have looked carefully through Dr. Verdi's *Progressive Medicine*, but have failed to find any new discoveries or new thoughts! His rules for hygiene and dietetics are as old as Moses; his medicinal treatment is a mixed chemico-physiologico-pathologico arrangement, which does not seem to be based on any sound reasoning, nor to be followed by even a tyro's success! There does not appear to be a spark of originality in the book; its ideas and methods have all been advocated before, notably by Dr. Kidd.

Our columns are open to Dr. Verdi if he cares to *briefly* point out some of those "new thoughts" he alludes to so grandiloquently in his Preface.

American Medicinal Plants. An Illustrated and Descriptive Guide, etc. By C. F. Millspaugh, M. D. Fascicle II, containing Nos. 6 to 10. New York and Philadelphia: Bœricke & Tafel. 1885.

We remember well the remark of one of our lecturers on Materia Medica, that the *country doctor* was expected to recognize any plant or weed his admiring fellow-citizens might chance to bring him for classification! If this be true, we feel sure these excellent plates Dr. Millspaugh is giving us will make such recognition easy.

We repeat again, these plates are simply beautiful, and to our eyes appear very accurate. Each fascicle contains plates of some *thirty* plants. *Six* of these beautiful plates for *one* dollar is certainly very cheap. For sale by Messrs. Bœricke & Tafel.

